# Darwinian perspectives on the evolution of human languages

**DOI:** 10.3758/s13423-016-1072-z

**Published:** 2016-07-01

**Authors:** Mark Pagel

**Affiliations:** 1 School of Biological Sciences, University of Reading, Whiteknights, Reading, RG6 6UR UK; 2 The Santa Fe Institute, Hyde Park Road, Santa Fe, NM 87501 USA

**Keywords:** Languages, Evolution, Darwin, Phylogeny

## Abstract

Human languages evolve by a process of descent with modification in which parent languages give rise to daughter languages over time and in a manner that mimics the evolution of biological species. Descent with modification is just one of many parallels between biological and linguistic evolution that, taken together, offer up a Darwinian perspective on how languages evolve. Combined with statistical methods borrowed from evolutionary biology, this Darwinian perspective has brought new opportunities to the study of the evolution of human languages. These include the statistical inference of phylogenetic trees of languages, the study of how linguistic traits evolve over thousands of years of language change, the reconstruction of ancestral or proto-languages, and using language change to date historical events.

Writing in his *Descent of Man* (1871), 11 years after the publication of the *Origin of Species* (1859), Darwin observed that “the formation of different languages and of distinct species, and the proofs that both have been developed through a gradual process, are curiously the same” (Darwin, 1871, p. 59; the German linguist Schleicher had made the same point in 1863, eight years before Darwin). As usual, Darwin (and Schleicher) was on to something, because it turns out that the transmission and evolution of genes and languages share a number of striking parallels (see Table 1).

**Table 1:** Some parallels between biological and linguistic evolution

Biological evolution	Language evolution
Discrete heritable units (e.g., nucleotides, amino acids, and genes)	Discrete heritable units (e.g., words, phonemes, and syntax)
DNA copying	Teaching, learning, and imitation
Mutation (e.g., many mechanisms yielding genetic alterations)	Innovation (e.g., formant variation, mistakes, sound changes, and introduced sounds and words)
Homology	Cognates
Natural selection	Social selection and trends
Drift	Drift
Speciation	Language or cultural splitting
Concerted evolution	Regular sound change
Horizontal gene transfer	Borrowing
Hybridization (e.g., horse with zebra and wheat with strawberry)	Language Creoles (e.g., Surinamese)
Geographic clines	Dialects and dialect chains
Fossils	Ancient texts
Extinction	Language death

Indeed, the transmission of linguistic information is not merely analogous to the transmission of genetic information: At a mathematical level, the two can be seen as formally equivalent, given certain simplifying assumptions. Both genes and languages can be represented as digital systems of inheritance, built on the transmission of discrete chunks of information—genes in the case of biological organisms, and words in the case of language. Genes in turn comprise combinations of the four bases or nucleotides (A, C, G, T) whereas words can be modelled as comprising combinations of discrete sounds or phones (in fact, phones or sounds vary in a continuous space, but languages are commonly represented as expressing a particular set of discrete phonemes).

The similarities between these two systems of inheritance raises the possibility that we can import to the study of languages ideas, approaches, and methodologies originally developed to investigate genetic systems—a prospect that has been fulfilled: In recent years a field of phylogenetic and comparative studies of how languages evolve has grown up around ideas and methodologies adapted from evolutionary biology and statistics (Pagel, 2009). I will describe some of these in this article, attempting to show how a Darwinian perspective has allowed researchers to use languages to test questions of human history as well to test questions of how languages themselves evolve.

## Phylogenies of languages

Linguists have known from at least the late 18th century (Jones, 1824) that languages evolve from earlier ancestral languages, eventually giving rise to family trees or what biologists call *phylogenies* of related contemporary languages.

**Fig. 1:**
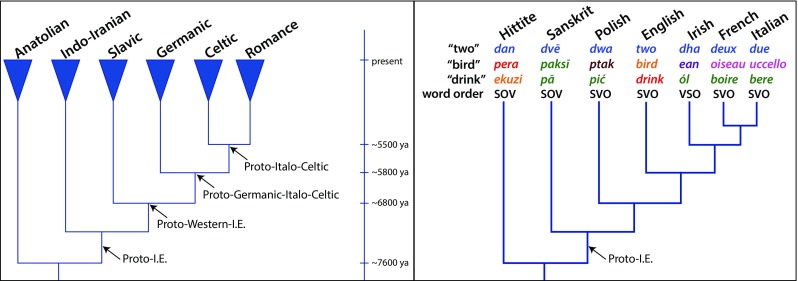
**a** A phylogeny of the Indo-European languages showing several of the major groups and the historical branching points. Triangles at the tips of the tree indicate groups of contemporary languages that share a common ancestor. The base or root of the tree corresponds to the ancestral or proto-Indo-European language that might have been spoken sometime around 6,000 (Chang, Cathcart, Hall, & Garrett, 2015) to 7,500 years ago (Bouckaert et al., 2013). Timings shown follow Bouckaert et al. (2013); **b** A phylogeny highlighting languages from each of the major groups in **a** and showing the word in that language for *two, bird,* and *drink*. Words represented with the same color are in the same cognate class, indicating they derive from a common ancestral word. The words for *two* are cognate across the entire Indo-European language family suggesting their common ancestor was present in proto-Indo-European. Words for *bird* are much younger, and *drink* is intermediate. “Word order” denotes the dominant ordering of subjects (S), verbs (V), and objects (O) in main clauses of a language. The presence of SOV in Hittite and Sanskrit—two languages that branched off early from the root of the tree—suggests that SOV is the ancestral state, and statistical modelling supports this inference (text)

A phylogenetic tree is a hypothesis about the specific sequence of historical branching events leading from a common ancestor forwards in time to the contemporary groupings, be they biological species or languages. One of the best studied linguistic phylogenies is that defined by the Indo-European (I-E) language family, a highly simplified form of which is shown in Fig. 1a.

Figure 1a depicts what is sometimes known as the Anatolian origin of the Indo-European languages, which scholars date to around 7,500 years ago (Bouckaert et al., 2012, 2013). Others (e.g., Chang, Cathcart, Hall, & Garrett, 2015) prefer a more recent origin of the I-E languages—closer to 6,000 years ago—and not in Anatolia but somewhere in the Russian steppes. Either way, the descendant languages of this family are now spoken widely across western Eurasia and the Indian subcontinent. Some of these modern descendants include the Celtic, Germanic, and Romance languages of western Europe, the Slavic languages of Russia and much of the Balkans, and the Indo-Iranian languages including Persian, Sanskrit, and many of the languages of the Indian subcontinent.

Phylogenies can seldom be observed directly because the ancestors that are inferred to have existed at the base or origin of the tree and then at its internal nodes or branching points (see Fig. 1a) no longer exist, having typically been replaced by their descendants over time. Even when ancient samples exist—possibly as fossils or as ancient texts—it is not always obvious precisely where on the tree they would be placed. For these reasons, phylogenies must be inferred, and this is usually accomplished by using information available in contemporary species or languages. Where biologists use the similarities and differences in genes among a group of species to infer biological phylogenies, linguistic phylogenies can be inferred from similarities and differences in lists (e.g., Swadesh, 1952) of common vocabulary words, or in patterns of shared sounds and sound use (Hruschka et al., 2015).

Modern phylogenetic studies rely on statistical models of evolution based on the principle of *likelihood* to guide the inference of phylogenetic trees (Edwards, 1972; Felsenstein, 2004; Pagel, 1999, 2000, 2009). The likelihood is defined as the probability of the data (the patterns of similarity and differences in genes or words) given a tree or phylogeny and a model of evolution that contains assumptions about how words evolve. The likelihood is conventionally written as

$$ L=P\left(\left.D\right|M,T\right), $$

where *L* is the likelihood, *D* stands for the data, *M* is the model of evolution, and *T* is the phylogenetic tree.

The model of evolution *M* is, in a linguistic setting, a mathematical-statistical statement about the rate at which new forms arise and change. If words are the raw data, then the model estimates the rate at which new unrelated words arise. If the raw data are the phonemes or sounds that make up the words, then the model estimates the rate at which these sounds change from one to the other (e.g., Hruschka et al., 2015).

Given a list of words shared among the various languages (e.g., Fig. 1b), the goal is to find the phylogenetic tree (including its branching patterns, the lengths of the branches of the tree, and the relative timings of events in the tree) that makes the data (the word lists) most likely or probable given the model of evolution. The assumption is that change is rare or slow, and so contemporary forms that are more similar are probably more closely related than forms with less in common. The statistical nature of the likelihood means that alternative hypotheses (alternative trees) can be tested against the “best” or most probable tree by comparing their likelihoods, allowing researchers to support some descriptions of history over others.

Statistical likelihood models have been used to reconstruct phylogenies of the Austronesian languages (Gray, Drummond, & Greenhill, 2009), the Indo-European languages (e.g., Bouckaert et al., 2012; Chang et al., 2015; Gray & Atkinson, 2003; Pagel, 2000), the Turkic languages (Hruschka et al., 2015), the Semitic languages (Kitchen, Ehret, Assefa, & Mulligan, 2009), Japonic (Lee & Hasegawa, 2011), Bantu (Grollemund, Branford, Meade, Venditti, & Pagel, 2015), and Arawak (Walker, Robert, & Ribeiro, 2011).

In addition to supplying descriptions of the history of a group of languages, language trees might be especially well suited to investigating questions of relatively recent human history, especially those of human migration. Gene evolution can be too slow to resolve recent events, and often there has been migration or intermarrying between groups that has diluted genetic differences even while cultural differences have been maintained. For instance, language trees have been used to study the timing and causes of the spread of Indo-European languages (e.g., Bouckaert et al., 2012; Chang et al., 2015; Gray & Atkinson, 2003), the pace of occupation of the Pacific by the Austronesian people (Gray et al., 2009), and the migration routes of the Bantu-speaking people through Africa (Currie, Meade, Guillon, & Mace, 2013; Grollemund et al., 2015).

Linguistic phylogenies are also routinely used to investigate questions of human cultural evolution. Here, language trees have been used to study the rise and spread of farming (Gray & Atkinson, 2003), the movement of ancient horseman from the Russian steppes (Chang et al., 2015; Haak et al., 2015), the evolution and spread of dairying (Holden & Mace, 2003, 2009; Mace, Jordan, & Holden, 2003), relationships between religious and political practices (Watts et al., 2015), changing political structures (Currie, Greenhill, Gray, Hasegawa, & Mace, 2010), and even the age of fairy tales (da Silva & Tehrani, 2016).

## Rates of word replacement

Linguists define two words as *cognate* if they descend from a common ancestral word, just as biologists define two genes as *homologous* if they descend from a common ancestral gene (see Table 1 and Fig. 1b). An intriguing feature of human languages is that the words for some meanings get replaced over the course of evolution by new unrelated or noncognate words far more frequently than the words for other meanings. For instance, the words used to denote the concept of *two* of something are cognate among all of the Indo-European languages whereas the words for *bird* or *drink* change more often (see Fig. 1b).

A phylogenetic perspective on this question immediately tells us that the related sounds for *two* trace their ancestry far farther back in time than the sounds for *bird*. But, if a word is just a sound that conveys a meaning, why is it that some meanings retain their words far longer than others? It is not the words themselves: *Bird* would be a perfectly fine sound to convey the idea of two objects, and *two* a perfectly fine sound to describe egg-laying, feathered animals that fly.

If words are coded as cognate or not among various languages, and this information is arrayed on a phylogenetic tree, as in Fig. 1b, it is possible to estimate the rates at which new cognate classes arise per unit time for different words. Applying statistical likelihood models comparable to those used for inferring phylogenies reveals a 100-fold difference in this rate in the Indo-European languages (Pagel, Atkinson, & Meade, 2007). Numeral words and pronouns (*I, you, who, two, three, five*) tend to be among the slowest evolving (Pagel et al., 2007; Vejdemo & Hörberg, 2016) whereas many adjectives and verbs (e.g., *dirty, rotten, wet, smell, squeeze*) have high rates of change. Nouns (e.g., *ear, foot, salt, egg, star*) often fall somewhere in the middle.

The differences in the rates of change among the various categories of words in Indo-European languages are replicated across many of the world’s languages (Pagel, 2009), suggesting a common cause. It turns out that the single best predictor of how long a word will last before being replaced by a new noncognate form is the frequency with which it is used in common everyday speech: Words that are used frequently tend to be replaced at a slower pace than those that are infrequently used (Pagel et al., 2007), and the frequency with which specific meanings are used seems to be much the same around the world (Calude & Pagel, 2011).

## Linguistic archaeology

Armed with knowledge of the rates at which words change, it should be possible to perform what could be called *linguistic archaeology*—namely, to plumb what the past might have been like or to estimate when certain events occurred. In this way, languages can be used to ask and test questions about human history.

Ancient languages seldom leave a fossil trace, and so historical linguists often attempt to reconstruct past or *proto*languages from the information in a set of related contemporary languages (e.g., Bomhard, 2008; Crowley & Bowern, 2010). The success of this endeavor depends directly on rates of change: Words that change slowly over long periods of time, such as the numeral words, might provide a clear signal of their past or ancestral states. For instance, the proto–Indo-European (see Fig. 1a, b) word for *two* might have been *duo*, and *tria* might have been the proto–Indo-European word for *three,* stretching back perhaps 7,500 years (Bouckaert et al., 2013).

We (Pagel et al., 2007, and above) were interested to see if we could go back even further in time by studying words with exceptionally slow rates of change. If these “ultra-conserved’ words exist more widely than in the Indo-European languages that we originally studied, cognate forms of them will be found today in a diverse range of languages that are the descendants of an even older common ancestor.

We found evidence for around 20 such ultra-conserved words, related forms of which can indeed be found today in languages from all over Eurasia (Pagel, Atkinson, Calude, & Meade, 2013). Among the ultra-conserved words were *thou* (*you*), *I, we, who, what, mother, bark* (of a tree), *ashes* and *fire.* As expected, the ultra-conserved words tended to correspond to meanings that are used at high frequency, or at least might have been in our distant past. By taking into account the rate of change of these words, we were able to posit the existence of an ancestral language that would have been spoken around 15,000 years ago, a time shortly after the last Ice Age, when all human groups were still hunter–gatherers.

Words can also be used to date historical events, an endeavor that Swadesh (1952) referred to as *glottochronology.* The Homeric epics are among some of the greatest masterpieces of literature. The *Iliad* is set during the Trojan War, the 10-year siege of the city of Troy (Ilium) by a coalition of Greek states, and so it must have been written sometime after the 12th century BCE—if indeed the wars were ever fought. But how much later? Was Homer effectively a “war reporter,” writing an account of events soon after they happened, or was he an historian?

Herodotus, writing in the *Histories*, Book II.53 around 450 BCE, remarked that Homer “lived, as I believe, not more than 400 years ago.” Many modern classicists and historians prefer a more recent, mid-8th century date for the Iliad. We (Altschuler, Calude, Meade, & Pagel, 2013) decided to try to estimate a date for the *Iliad* by investigating patterns of cognacy among the 200 words of Swadesh’s (1952) fundamental vocabulary in three languages: Modern Greek, Homeric Greek from Homer’s *Iliad*, and Hittite, a language distantly related to both modern and Homeric Greek.

We first recorded whether each word in the Swadesh list was cognate or not between pairs of the three languages. Then, we solved for the date in history that was the most likely for the *Iliad,* given our knowledge of the rates of change of the words and the patterns of cognacy we observed. Our calculation suggested that the original text of the *Iliad* was released in approximately 762 BCE. This date is in close agreement with classicists’ and historians’ beliefs arrived at independently by studying historical references and the nature of Homeric Greek as expressed in the *Iliad.*

## The structure of languages

One way linguists classify languages is by their structural properties of syntax, grammar, and word morphology (e.g., Longobardi & Guardiano’s, 2009, study of syntax). A well-known structural feature of a language is the order of the words in its sentences. The sentence “I kicked the ball,” for example, is SVO, or subject (S), verb (V), object (O). Of the six possible orderings of subjects, verbs, and objects in a sentence, two—SVO and SOV—dominate the world’s languages; two others—VSO and VOS—account for ~10 % of languages; and the remaining two—OSV and OVS—are rare (Dryer & Haspelmath, 2013; Gell-Mann & Ruhlen, 2011; Greenberg, 1963).

A phylogenetic-statistical perspective makes it possible to trace the history of this important structural feature of languages, including inference of the most probable ancestral or founding word order for a group of languages. First, each of a number of related languages must be evaluated for its word order, and these are then arrayed on a phylogeny of the languages, as in Fig. 1b. With this combination of data and phylogenetic tree, it is then possible to study the evolution of the changing word orders in much the same way as I described in a previous section for studying rates of evolution of cognate classes.

Applying this statistical approach to the Indo-European languages suggests that SOV (“I the ball kicked”) was the most probable word order of the ancestral or proto–Indo-European languages (Dunn, Greenhill, Levinson, & Gray, 2011; Pagel, 2009), and SOV is also inferred to be the likely ancestral state of many other language families (Gell-Mann & Ruhlen, 2011; Maurits & Griffiths, 2014).

Happily, the phylogenetic-statistical inference of the ancestral word order agrees with earlier conclusions from linguists (e.g., Lehmann, 1981). But we might then ask what the phylogenetic-statistical perspective brings or adds in this situation. Two answers might be given. One is that an approach using phylogenetic information can lead to different conclusions from those that fail to account for the relatedness among languages. Imagine, for example, that one investigated the languages of Fig. 1b without regard to their phylogeny. A simple count that ignores any information on the relatedness of the languages might suggest that SVO was the ancestral word order.

A second reason to use the phylogenetic-statistical approach is that it can locate on the tree the timings and placement of specific changes, and it can estimate both the dominant directions and the rates at which various transitions occur (see Maurits & Griffiths, 2014; Pagel, 2009). Pagel (2009), for instance, found that SOV routinely gave rise to SVO word orders, and SVO to VSO, but that reverse transitions were rare or nonexistent.

## The nature of evolutionary changes

Darwin’s quote at the outset of this article is often used to illustrate his attachment to a gradual or smooth view of evolutionary changes. But two phenomena—punctuational change and concerted evolution—give slightly different perspectives on this gradual mode of evolution.

Phylogenetic trees for Austronesian, Bantu, and Indo-European languages all show that languages with a rich history of language-splitting events have diverged more from their ancestral languages than extant languages with fewer splitting events in their pasts (Atkinson, Meade, Venditti, Greenhill, & Pagel, 2008). It is as if in Fig. 1a we would expect more linguistic change to have occurred along the path from the root of the tree up to the Celtic or Romance languages than we would along the path from the root to the Anatolian, Indo-Iranian, Slavic, or Germanic languages. The amount of time is the same in every case, but they differ in the number of splitting events (this example is hypothetical because the tree in Fig. 1a and b only incudes a few of the languages along each path). We observed a similar pattern for genetic evolution among biological species (Pagel, Venditti, & Meade, 2006).

The explanation for the increased amounts of evolution along branches with more splitting events seems to be that at times of “speciation” and of “lineage splitting”—when new languages or species form—a short episode or punctuational burst of evolution occurs. Elsewhere, we have suggested that speciation and cultural splitting are special times of evolution when multiple factors can come into play that accelerate the pace of change (Venditti & Pagel, 2010, 2014). Anthropological accounts of indigenous societies suggest that at times of cultural change, new groups will often actively change their language to distinguish themselves from their neighbors (Pagel, 2012).

Evolutionary biologists use the term *concerted evolution* to describe the strange phenomenon of a nucleotide replacement (one nucleotide being substituted for another) at a specific site in one gene, being quickly followed by the same nucleotide replacement at the same site in other, typically related, genes. A form of concerted evolution known as *regular sound change* is also observed in languages, where a specific phoneme or sound changes to the same other phoneme in many words in the lexicon (Crowley & Bowern, 2010; Hruschka et al., 2015). A well-known example is the *p*→*f* sound change in the Germanic languages, where an older Indo-European *p* sound was replaced by an *f* sound, such as in *pater*→*father*, or *pes, pedis*→ *foot.*

As with punctuational episodes of change, concerted evolution is not what we normally associate with the gradual, even plodding, pace of evolution. On the other hand, neither punctuational nor concerted change violates a Darwinian view of evolution. Both are simply instances in which the pace of evolutionary change increases, sometimes dramatically, for short periods of time.

## Discussion

Where biological bodies are the temporary repositories of genes, human minds are the temporary repositories of words. Both genes and words increase their probability of being transmitted—one into a new body, the other into a new mind—by adopting forms that are fitter than their competitors. The last 50 years or so of evolutionary studies has documented countless instances of the adaptation of genes. Now, in the last 10 to 20 years, the increasing use of evolutionary perspectives in combination with phylogenetic-statistical methods is documenting patterns in the evolution of languages, words and sound systems that are consistent with language adapting to the minds and habits of its speakers (Christiansen & Chater, 2008). These new methods bring an explicit hypothesis testing rigor and make possible inferences, analyses, and tests not available to traditional studies.

Most of the preceding discussion has referred to patterns of evolution observed among languages. It is also possible to identify adaptive evolution occurring within single languages, that is, within a population of speakers. For instance, it is well known that frequently used words are shorter—the easier to say—conforming to Zipf’s principle of least effort (Zipf, 1949). Rates of linguistic change also appear to be faster in larger populations (Bromham, Huaa, Fitzpatrick, & Greenhill, 2015), confirming a key prediction of adaptive evolution: In larger populations, selection is better able to overcome the effects of random drift.

Where the transmission of genes has been responsible for the diversity of organismic life over the last 3.5 billion years, the transmission of sounds and words has been responsible for the diversity of languages over the approximately 160,000 to 200,000 year history of our species. But in that relative blink of an eye, the transmission of linguistic information—a feature confined to human society—has been far more influential on the recent history of the world than have genes. It could even be said that language’s role in the transmission of the information that makes our societies possible—the development and continued improvement of nearly all of our artefacts and technologies—means that genes have now been superseded by this new but powerful Darwinian replicator.
